# Relict subduction initiation along a passive margin in the northwest Indian Ocean

**DOI:** 10.1038/s41467-019-10227-8

**Published:** 2019-05-21

**Authors:** Dhananjai K. Pandey, Anju Pandey, Scott A. Whattam

**Affiliations:** 1 ESSO-National Centre for Polar & Ocean Research, Vasco da Gama, Goa, 403804 India; 2 Department of Geosciences, King Fahd University of Petroleum and Minerals, Dhahran, 31261 Saudi Arabia

**Keywords:** Geochemistry, Geodynamics, Geology, Tectonics

## Abstract

The tectonic evolution of Laxmi basin, presently located along western Indian passive margin, remains debated. Prevailing geodynamic models of Laxmi basin include two mutually competing hypotheses, culminating in either a hyper-stretched continental crust or an oceanic crust overlying an extinct spreading centre. The longstanding conundrum surrounding its precise crustal affinity precludes a complete understanding of the early opening of the Indian Ocean. Here, we present distinct geochemical and geophysical imprints from the igneous crust in Laxmi basin obtained through International Ocean Discovery Program Expedition 355. The geochemical and isotopic signatures of the Laxmi basin crust exhibit uncanny similarities with forearc tectonic settings. Our observations imply a relict subduction initiation event occurred in the Laxmi basin in the Late Cretaceous-Early Cenozoic that marks a significant Cenozoic plate reorganisation record in the northwest Indian Ocean. New findings therefore warrant re-evaluation of the Gondwana breakup to account for the nascent subduction in the northwest Indian Ocean.

## Abstract (web summary)

The tectonic evolution of the Laxmi basin, in the northwest Indian Ocean, remains controversial. Here, the authors present new geochemical and seismic data which indicate the Laxmi basin formed by a subduction initiation event during the break-up of Gondwana.

## Introduction

**Fig. 1:**
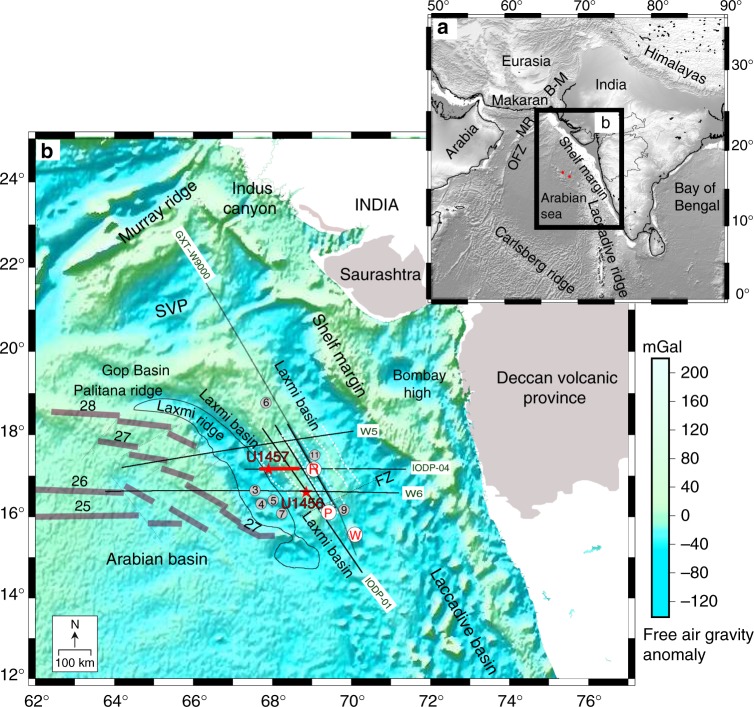
Tectonic overview of northwest Indian Ocean. A regional geological overview (**a**) and a detailed structural map (**b**) of western Indian passive margin depicting location of Laxmi basin in the northwest Indian Ocean. Satellite derived free-air gravity anomalies^55^ are superimposed on the map. SVP Saurashtra volcanic province, MR Murray ridge, R Raman seamount, P Panikkar seamount, W Wadia guyot, B-M Bela-Muslim Bagh Ophiolite belt, OFZ Owen Fracture Zone^7^. Open numbers besides grey bands represent magnetic anomalies in the Arabian basin^19^. Solid, thin lines (in black) show regional seismic profiles from present and previous studies respectively. Thick, red part of the E–W oriented seismic profile IODP-04 is displayed in Fig. 2. Grey circles with numbers mark locations of refraction seismic experiments^6^. Expedition 355^26^ sites (U1456 and U1457) are marked in red colour. Magnetic lineations in Laxmi basin^18^ are shown on the map (white broken lines)

Passive continental margins with their vast global extent offer unique opportunities for exploring continental extension and crustal growth. The western passive margin of India inherits vital imprints of the Gondwana break-up and early evolution of the Indian Ocean, yet it is one of the least explored continental margins^1–4^ (Fig. 1). Complex rifting accompanied by anomalous magmatism^1,2^ make it significantly different from both, classic magma-rich or magma-poor margins^2^. Initially, India and the Seychelles-Laxmi ridge (SLR) conjoined blocks separated from Madagascar through seafloor spreading in the Mascarene basin at ~84 Ma^5–14^ (Fig. 1). Subsequent northward drift of greater India (~80–90 mm/year)^7,11^ was complemented by highly complex sinistral transform and rotational motion^15–17^. Contemporaneous asymmetric seafloor spreading, transpressions and multiple ridge reorganisations produced a triangular geometry in the Mascarene basin and forced the India-SLR conglomerate to rotate counter-clockwise as it drifted away from Madagascar^15–18^. Late Cretaceous plate kinematic reconstructions^7–11^ suggest that the Indian plate was surrounded by spreading ridges, transform faults and fracture zones^15–24^ in the south, subduction in the north^24,25^ and ophiolite emplacement along its northwestern edge^15–17,23,24^. Between approximately 80 and 65 Ma, the Indian plate drifted rapidly away (~150–160 mm/year) prior to India-Eurasia collision (~50 Ma)^25^. A major ridge-reorganisation at ~62 Ma^19–22^ in the northwest Indian Ocean heralded seafloor spreading between the Seychelles and Laxmi ridge (LR) and effectively marked the onset of formation of the proto-Carlsberg ridge (Fig. 1). The Seychelles-LR separation is considered as contemporaneous to Deccan volcanism (~68.5–60 Ma with peak magmatic activity around ~65 Ma) when the Reunion hotspot impinged beneath a waning Indian lithosphere^3,4,13^. However, accurate estimates of the extent of offshore Deccan volcanism also remains elusive^26^. Highly complex northward journey of India at ~80–65 Ma produced numerous enigmatic structures along its western passive margin, e.g., the Laxmi basin (LB), Gop rift and numerous seamounts^13,14,20,21^ (Fig. 1). Decisive information about the LB is vital for deciphering the exact sequence that led to Gondwana breakup and subsequent rift-to-drift tectonics in the Indian Ocean. Furthermore, during this breakup the LB likely remained proximal to the Deccan volcanic province (DVP)^3,4^ and therefore may offer an exquisite opportunity to investigate concurrent plume-rift interactions^4,5^ in the Indian Ocean (Fig. 1).

The LB is a ~300 km wide, marginal depression enclosed by Indian continental shelf and the LR on either sides (Fig. 1). The E–W trending Gop rift lies obliquely north of the LB across a fracture zone^7,17–23^. Published marine geophysical studies confirm that the NW-SE trending axial segment within the LB also contains isolated seamounts (Fig. 1) with high relief and flat tops with diameters of up to ~20 km (Wadia, Raman and Panikkar Seamounts)^12,14,18^. These seamounts, with no precise ages, are flanked by flexural moats at their bases implying considerable post-emplacement subsidence^26^. The LB displays an overall positive free-air gravity anomaly (implying thin crust) with distinct axial lows (~20 mgal) aligned with axial segments (Fig. 1). High-resolution seismic reflection and bathymetry data from LB evince rugged and substantially deformed basement between a variably thick sediment apron and a magmatically modified lower crust^12,14,22,26^. The igneous basement in the LB is generally shallower (~5–6 s two-way travel time—TWT) than normal oceanic crust to its west in the Arabian Sea (~7.5 s TWT)^26^. Bhattacharya et al.^18^ and subsequent studies^7–14,20–22^ identified magnetic lineations and transform faults in the LB with varying rift durations (C33n-C28n-C33n^18^ sequence to C30n-C25r-C30n^20^ or C29n-C28n-C29n^21^) and thus favoured an oceanic affinity of the crust with an underlying extinct spreading centre (see lineations in Fig. 1). However, the extent of magnetic lineations as well as inferred rift durations in LB vary profusely from one study to another, indicating a pre-Deccan, ultra-slow^18^ (~10 mm/year; i.e. ~79.5–62.5 Ma)^18^ to slow^20^ (~20 mm/year; ~67.6–56 Ma), to post Deccan, reasonably fast spreading^21^ (~30 mm/year; ~64.7–62.5 Ma). Recent studies^12,14^ offer alternative interpretations to these magnetic lineations^18^ by attributing them to possible magmatic intrusions during extensional tectonics. In view of numerous equivocal interpretations, we consider the interpretations of Bhattacharya et al.^18^ for the present work to avoid an out of scope discourse. Interestingly, in view of proposed magnetic lineations^18^, emplacement of axial seamounts over highly attenuated crust could be younger than Anomaly 28 (~62 Ma). Contrary to expectations, available seismic images neither corroborate a characteristic axial valley nor a relict magma chamber clearly underneath an axial segment, i.e., the Panikkar ridge^26^. The crust underneath the LB appears to be ~7–12 km thick (*V*_p_ ~ 4.7–7.4 km/s)^6,12,14^ with lower (7.6–7.8 km/s) than standard oceanic Moho velocities (Supplementary Fig. 1). High heat flow measurements in the LB (~57–60 mW/m^2^)^26^ are compatible with ~79–63 Ma old oceanic crust^27^, consistent with putative ages of the lineations^18^. However, shallower basement in the Laxmi basin is in variance with predicted plate cooling models^27^.

In contrast, the adjoining LR is a ~700 km long, enigmatic, arcuate structure with overall NW-SE strike before it swerves E–W at ~19° N near the Palitana ridge (Fig. 1). As opposed to the LB, the LR displays broad negative free-air gravity anomalies (−50 mgal) indicating thicker than normal oceanic crust beneath^12^. New seismic images reveal an irregular basement underlying a thin sediment apron^26^ beneath the LR. Basement deformation and numerous late stage magmatism in its vicinity suggest its formation in a magma-rich environment^14,22^. To date, the Moho boundary beneath the LR has not been imaged, however, crustal modelling experiments infer an attenuated crust^12,14^ (~16–20 km thick, *V*_p_ = 4.3–7.2 km/s)^6,12,14^ with high velocity magmatic underplating (*V*_p_ ~ 7.4 km/s)^12,14^ of the lower crust (Supplementary Fig. 1). Some consider the LR as a continental sliver^6,9^ or an abandoned spreading ridge^22^. In order to dispel inherent ambiguities across a myriad of proposed geodynamic models, physical sampling of the LB crust became essential. Here, for the first time, we evaluate genesis and growth of the LB using petrological, geochemical and isotopic analysis of igneous basement retrieved through International Ocean Discovery Program (IODP) expedition 355^26^. Our new results suggest that the LB crust is similar to that observed in forearc settings formed during incipient subduction.

## Results

### Scientific drilling in the Laxmi basin

**Fig. 2:**
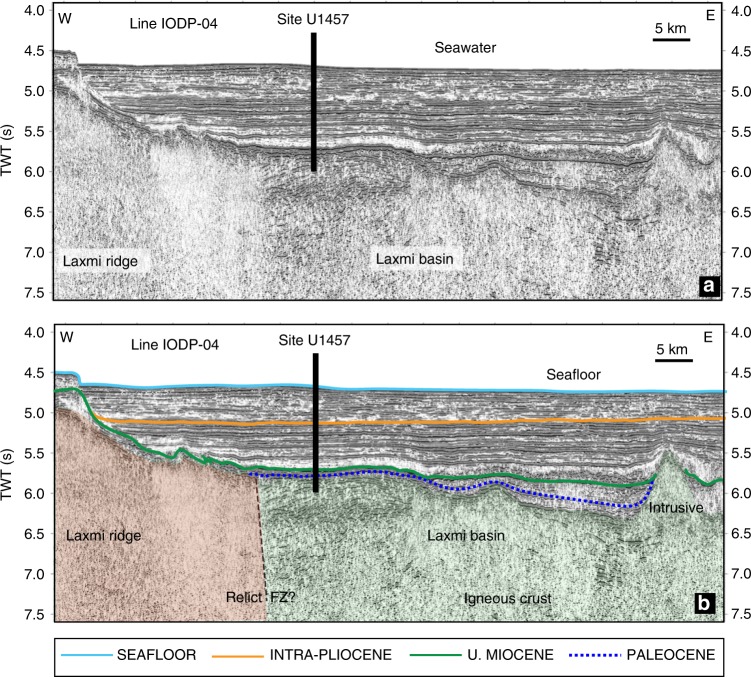
Seismic structure beneath the Laxmi basin. Location of IODP drill Site U1457 along the regional seismic profile IODP-04. The uninterpreted (**a**) and interpreted (**b**) sections are presented. Site U1457^26^ is strategically located between the Laxmi ridge and Laxmi basin. The igneous basement in Laxmi basin was reached at ~1090 m below seafloor (mbsf). Overlying Cenozoic sedimentary horizons are marked on the interpreted section. TWT (s)—two-way travel time. A 30 m thick early Paleocene^26^ unit (~63 Ma) overlies the igneous basement. Additional crustal age constraints are gathered from published magnetic anomalies^18^. Laterally coloured igneous basement section represents crustal segments corresponding to asymmetrically thick crusts on either sides, having different affinity and ages^18^. Based on seismic reflection characters and available magnetic age constraints, we propose a potential weak zone (FZ), which may have been responsible for inferred transient SI

Site U1457 (17°9.95′N, 67°55.81′E) drilled and cored during IODP Expedition 355 in the LB is located at ~400 km and ~800 km off India and Pakistan coasts, respectively. This site was strategically chosen between the LB and the LR^26^ where different crustal types are gelled across possible fracture zones/transform faults (Fig. 2). Sixty days of drilling and coring operations onboard JOIDES Resolution in the LB commenced below ~3600 m of water depth and penetrated ~1090 m of Cenozoic sediment plus ~16 m of igneous basement for the first time^26^. A ~30 m thick volcaniclastic unit overlies igneous basement at Site U1457 and comprises claystone, siltstone and volcaniclastic breccias with sparse manganese and carbonate nodules^26^. Core samples 355-U1457C-93R-1, 128 cm, through 94R-CC (1063.48–1073.69 m below seafloor) contain an early Paleocene assemblage^26^. Biostratigraphic estimates confidently suggest an age ~63.3 Ma^26^ for the early Paleocene sediments directly overlying igneous basement thereby constraining the upper age bound of LB basalts (~63 Ma) (Fig. 2). The volcaniclastic breccias are of varying sizes and often with sharp and erosive bases. Light minerals, e.g., feldspar, mica and clay are abundant in this unit; volcanic glass is present in trace quantities and heavy minerals are absent. X-ray diffraction (XRD) data from the Paleocene unit confirm the presence of smectite, commonly attributed to physical breakdown of volcanic rocks^26^. An age-depth model from Site U1457 reveals several hiatuses with the longest one lasting up to ~50 Myr between the early-mid Miocene and the early Paleocene^26^.

Detailed shore-based petrological, geochemical and isotopic analyses of 35 basalt samples from Site U1457 spanning the entire basement section are discussed here (see the Methods section). Petrologically, LB lavas dominantly comprise plagioclase, clinopyroxene and olivine. Texturally, aphyric lava predominate and consist of microlitic plagioclase, clinopyroxene and euhedral to subhedral altered phenocrysts of olivine (Supplementary Fig. 2a). Micro olivine and clinopyroxene crystals are interserted within plagioclase laths. The phyric lava displays glomerocrysts of clinopyroxene, plagioclase and olivine set in a groundmass of microlitic plagioclase laths and clinopyroxene (Supplementary Fig. 2b). Occurrence of extremely large (~1–2 mm) plagioclase laths is distinctly observed in two samples (355U1457C96R3-15/19 and 355U1457C97R2-46/50 at 1092.48 and 1101.2 mbsf, respectively). These mega-plagioclase laths enclose olivine and display resorption of clinopyroxene suggesting their crystallisation after these mafic minerals. The groundmass is identical to the rest of the LB lava (Supplementary Fig. 2c). Typical occurrence of clinopyroxene and altered olivine grains before plagioclase in LB lava demonstrate that the likely crystallisation sequence was olivine–clinopyroxene–plagioclase. Mineral chemical data of plagioclase from these lavas point towards their highly calcic nature having anorthite mole % values up to 90.

### Geochemical imprints of the igneous crust in Laxmi basin

**Fig. 3:**
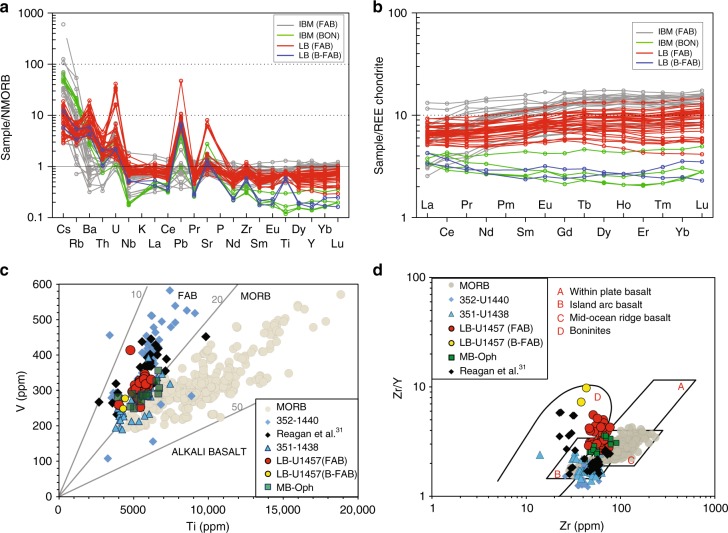
Geochemical discrimination diagrams of Laxmi basin lavas. Variations in LILEs and HFSEs abundances within the cored igneous section at Site U1457. **a** N-MORB normalised plot^53^. **b** Chondrite-normalised REE diagram^54^ showing compositions of Site U1457 lava. **c** Ti vs. V discrimination diagram^38^ showing Site U1457 lava compositions. Data sources for comparisons: Muslim Bagh-Bela ophiolites^43–46^, MORB^29,30^, 351^32^, 352^36^. **d** Zr vs. Zr/Y diagram^66,67,70^ for the Site U1457 lava. Most of the LB basalts fall into the arc and boninites fields. IBM Izu–Bonin Mariana, FAB forearc basalt, B-FAB boninitic forearc basalt, MB-Oph Muslim Bagh ophiolites

The LB basalts having SiO_2_ (48–50 wt%), MgO (8–9.5 wt%), Al_2_O_3_ (8–12 wt%) and FeO (8–9 wt%) along with trace element ratios such as Nb/Y (<0.9) and average Zr/TiO_2_ (~7) classify as sub-alkaline, tholeiitic basalt^28^. Although bulk major element compositions of the LB basalts are comparable to mid-ocean ridge basalt (MORB)^29,30^, they have distinctly low TiO_2_ and incompatible trace element compositions. The geochemical imprints of LB lava are prima facie akin to island arc signatures, e.g., depletion in high-field strength elements (HFSE, e.g., Nb, Th, etc.) and enrichment in large ion lithophile elements (LILE, e.g., Cs, Rb, Ba, Sr) (Fig. 3a). Such peculiar characteristics of LB lava encouraged us to compare them with known arc environments. Basalts displaying major element chemistry of MORB with moderate HFSE depletion and LILE enrichment have been extensively studied in the Izu–Bonin Mariana (IBM) subduction system. Reagan et al.^31^ classified such basalts as forearc basalt (FAB). The FABs are believed to have formed through decompressional and minor fluid-flux melting of the mantle around the leading edge of the Pacific plate during initial stages of subduction^32–34^. Therefore, identification of FABs on a margin implies the onset of a protoarc. Such successions typically begin with FAB and progress upwards to lavas with more depleted compositions before final highly depleted, boninites^31,33^.

Detailed comparisons of N-MORB normalised HFSE and LILE profiles of LB basalts and FAB types from IBM display striking resemblances (Fig. 3a). Most of the LB lavas show light rare earth element (LREE) depleted patterns except for the two petrographically distinct samples as discussed above. These two samples exhibit U-shaped total REE patterns suggestive of their extremely depleted nature (Fig. 3b). The classic U-shaped REE patterns are analogous to boninites that overlie the FAB^31^ sequence in the IBM subduction system as well as other boninites found elsewhere. Genesis of such a magmatic suite is attributed to the partial melting of a highly depleted and hydrated mantle in a forearc setting^35^. However, despite the characteristic U-shaped REE trends, the major element composition of these two LB samples do not strictly adhere to the IUGS boninite classifications (i.e., SiO_2_ > 52 wt%, MgO > 8 wt% and TiO_2_ < 0.5 wt%) and low, medium and high-silica boninite classifications^36,37^. In view of this, we prefer to denote these two transitional samples as boninite-like or boninitic-FAB (B-FAB). The FAB and B-FAB type lavas from Site U1457 are clearly distinguishable in N-MORB normalised plots where B-FAB exhibit highly depleted Nb and heavy REE (HREE) trends relative to the FAB (Fig. 3a). Ti/V ratios^31,37,38^ can also distinguish FAB from MORB. The B-FAB lava overlie the FAB sequences. The Ti/V ratio is crucial as it gauges oxygen fugacity, which is lower in subduction-modified mantle than MORB mantle^38^. Accordingly, the lower the Ti/V ratios, the higher the influence of water in magma-genesis^39,40^. On a Ti vs. V diagram^38^, Site U1457 lavas distinctly exhibit arc-like Ti/V ratios (~10–20) akin to IBM-FAB (Fig. 3c).

Igneous rocks from Site U1457 can be clearly classified as FAB and B-FAB with categorical subduction inputs. Elevated concentrations of slab-derived flux (Cs, Rb, Ba, Th, U, K, La, Ce, Pb, Sr) are superimposed on conservative mantle-wedge derived components (Nb, Zr, Sm, Eu, Ti, Dy, Y, Yb, Lu). This renders the characteristic spikes and troughs patterns of LB lava analogous to that of supra-subduction zone (SSZ) basalts (Fig. 3). Low trace and REE concentrations of the LB magma compared with N-MORB suggest a relatively depleted source. A depleted source is also evident on a Zr vs. Zr/Y diagram (Fig. 3d). The HFSE elements (e.g. Nb, Ta,Th, Zr, Hf and Ti) can offer additional constraints on the source mantle as they exhibit magmaphile behaviour during their genesis. Their ratios are good geochemical proxies for ascertaining the degree of partial melting^31,38^. Strong positive correlation between the refractory element pairs (Zr and Hf) in LB lava is clearly in sync with other SSZ environments (Supplementary Fig. 3a). Similarly, on a Nb/Yb–Th/Yb discriminant diagram^38,41,42^, the LB lava plot above the MORB array with Th/Yb ratio displaced to higher values, and overlap that of the IBM-FAB indicating a subduction influence (Supplementary Fig. 3). Overall, the observed geochemical markers in the LB lava consistently imply a subduction fluid-fluxed, depleted source characteristic to that of forearcs.

**Fig. 4:**
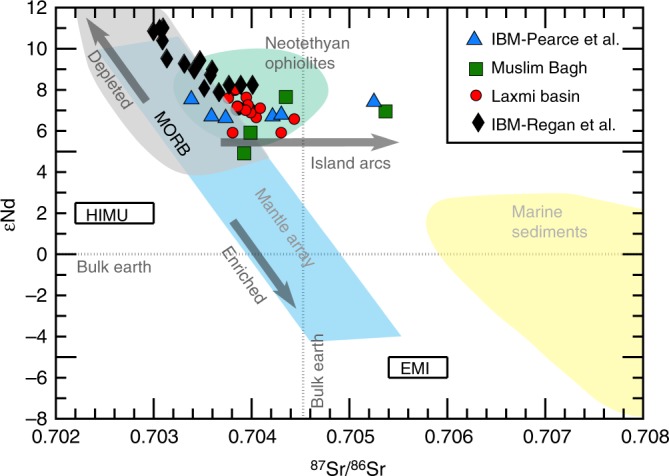
Radiogenic isotope ratios of Laxmi basin lavas. Plot shows ^87^Sr/^86^Sr versus ɛNd parameter for LB lava (age corrected for in-growth to pre-Deccan time ~66 Myr ago). Symbols as labelled on the diagram. Reference domains are adopted from published literature (MORB^68^, Neothethyan ophiolites^69^, IBM^31,33,34,41,42^, Muslim Bagh^44,45^)

Numerous studies have demonstrated that FAB are commonly observed in ophiolites, thereby suggesting an inherent link between subduction onset, forearcs and ophiolites^41,42^. The closest SSZ ophiolitic exposures adjoining LB are located at ~1000 km north i.e., the Muslim Bagh-Bela ophiolites^17,43–45^. These Neothethyan ophiolites are dated to have been emplaced upon the NW Indian margin between ~65 and 70 Ma^17,46^ along the northwestern continental margin of India (Fig. 1). Petrological, geochemical and isotopic comparisons of LB lavas with those from the Muslim Bagh-Bela ophiolites show strong coherence between the two (Figs. 3, 4 & Supplementary Fig. 3). Their proximal locations at the time of their emplacement as well as their compatible geochemistry prompts us to envisage a southward extension of this SSZ setting possibly to the proto-LB. Previous studies from the Central American Arc^41^ and IBM^42^ confirm that ophiolite-like forearc complexes—linked to subduction initiation events—may stretch for several thousands of kilometres today (~1200 and 1800 km, respectively). Therefore, our proposition of linking the proto-LB to the Muslim Bagh-Bela SSZ belt in view of their identical geochemical and geo-chronological context (see below) is tenable.

### Radiogenic isotopic characteristics of the Laxmi basin crust

The radiogenic isotope (^87^Sr/^86^Sr–^144^Nd/^143^Nd) systematics from Site U1457 were examined to characterise the LB basalts and its potential mantle sources (see the Methods section). Measured ^87^Sr/^86^Sr and ^144^Nd/^143^Nd ratios in the LB lava range between 0.703766–0.704439 (±0.000013) and 0.512940–0.513048 (±0.000016), respectively. Age corrected (~66 Ma) εNd values vary between +5.9 and +8.0. On a ^87^Sr/^86^Sr–ɛNd diagram (Fig. 4), the MORB and the OIB usually follow an anti-correlation trend along the mantle array whereas the depleted and enriched melt vectors, in general, lay juxtaposed in upper and lower parts of this diagram, respectively. The ^87^Sr/^86^Sr–^144^Nd/^143^Nd isotopic compositions of LB lava follow an analogous trend to the IBM-FAB domain confirming the SSZ affinity of the former (Fig. 4). Isotopic signatures of the LB crust also overlap with Indian MORB and Neothethyan ophiolite types (e.g., Muslim Bagh), suggesting their genetic link. Intersection of the arc and the mantle array trends around the lower half of the MORB domain on this diagram suggest potential subduction in a marginal setting (Fig. 4). Relatively higher ɛNd spread (~5.9–8) and less ^87^Sr/^86^Sr scatter in LB lava (~0.703766–0.704439) as compared with MORB suggests lack of significant contribution from enriched lithospheric mantle^35,40^. However, enhanced contributions from slab fluid derived flux is observed on the Ba/Th versus Th plot (Supplementary Fig. 3). Thus, the key geochemical and isotopic characteristics of LB lava succinctly favour a depleted and oxidised mantle source as compared with MORB.

## Discussion

**Fig. 5:**
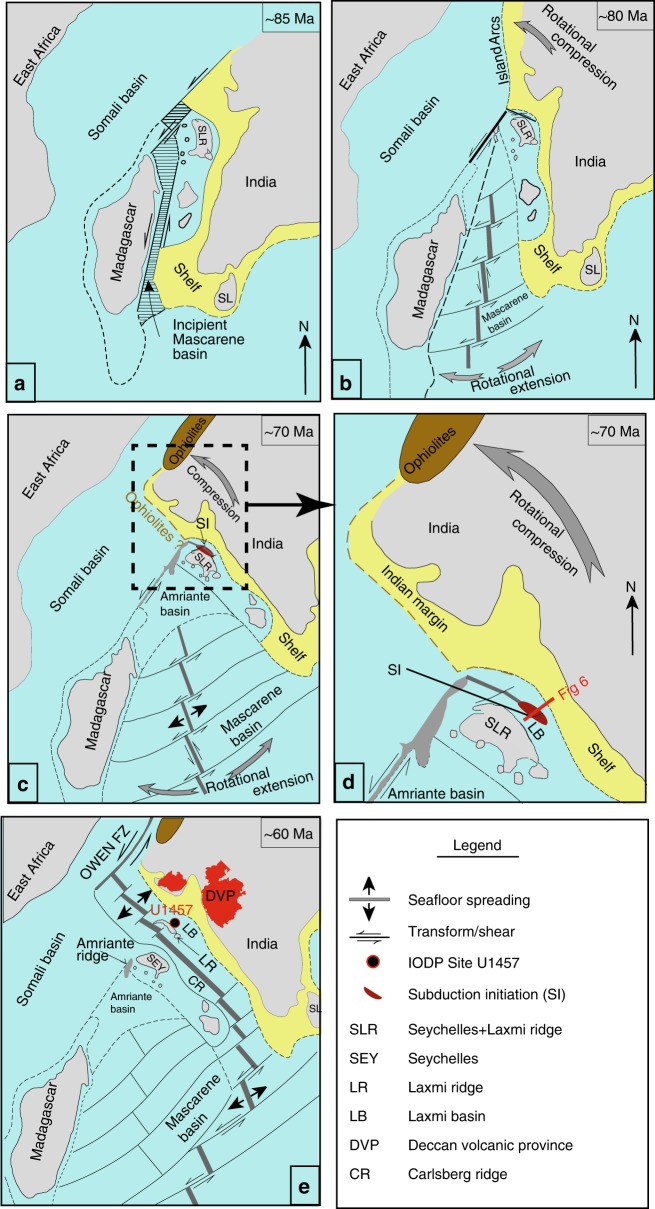
A generalised plate reconstruction model. The diagram depicts the late Cretaceous geodynamic evolution (not to scale) of northwest Indian Ocean^15–17,23^. **a** ~85 Ma: Sinistral transform motion between Madagascar and Seychelles-Laxmi-ridge-India conglomerate resulted in axial spreading and formation of incipient Mascarene basin. **b** ~80 Ma: Creation of oceanic crust in Mascarene basin induced an E–W separation across N–S translating plates. The asymmetric spreading rendered triangular geometry within the Mascarene basin and forced the Seychelles-Laxmi ridge-India conglomerate to rotate counter-clockwise as it drifted away from Madagascar. Further spreading and translational motion accompanied by simultaneous ridge reorganisation caused trans-tensional motion between Seychelles + Laxmi ridge conjoined block and India, producing a spreading axis segment along proto-Gop-Laxmi basin. **c** Continued rotational compressions led to subduction initiation (SI) and forearc magmatism in the Laxmi basin and island arc formation, volcanism and ophiolite complexes along the northwest Indian margin. **d** A zoomed in view of (**c**) shows the proposed location of SI. **e** Later, subduction failed in the Laxmi basin as a result of slab rollback and collision. Subsequent northward dextral motion of Indian plate led to its present day position. The Deccan volcanism (shown in red) occurred while Indian plate interacted with the Reunion plume during its northward journey (~65 Ma)^3^. Eventually, rifting moved out of Laxmi basin to the west of Laxmi ridge that separated Seychelles from Laxmi ridge that heralded modern Carlsberg ridge (~62 Ma)

It is significant that prevailing models of the genesis and evolution of the western Indian passive margin are solely driven by surface geophysical observations. Majority of such models focus on extensional tectonics in view of widespread marine magnetic anomalies^18–23^ and ancillary geophysical observations from the northwest Indian Ocean. The role of contemporaneous compressional tectonics on abutting margins has been often underestimated (Fig. 5). The first ever direct geochemical observations through IODP drilling at Site U1457 in the LB presented here, defy previous hypotheses and most of them now do not seem viable. Discovery of forearc crust in the LB unequivocally implies role of subduction initiation (SI)^31,36,41,42,47–49^ in its genesis and evolution (Fig. 5). Striking resemblance between geochemistry and isotopic signatures of the LB lavas and the IBM-FAB, encourages us to confidently conclude that a segment of the LB crust was formed upon SI. However, the present crustal configuration of the LB exhibits no signs of an ongoing subduction nor one in the recent past. This potentially leads us to infer that the proposed SI did not mature enough to attain a self-sustaining subduction system. Corroborating evidence gathered through the limited igneous stratigraphic sequences at Site U1457^26^ are also consistent with this proposition. Therefore, subduction in the LB may have been aborted shortly after its initiation, as mature magmatic arcs require a much longer gestation period (>10 Myr^47,50^) to produce IBM-like sequences^31,41,47^. The inferred time-frame of SI in Laxmi basin is comparable to other known SI events (e.g. IBM and Semail subduction systems where SI lasted for about 5–7 Myr^34,41^ before formation of a stable magmatic arc). Relatively shorter duration of SI in LB can be attributed to the absence of an established magmatic arc, which typically postdates the FAB.

**Fig. 6:**
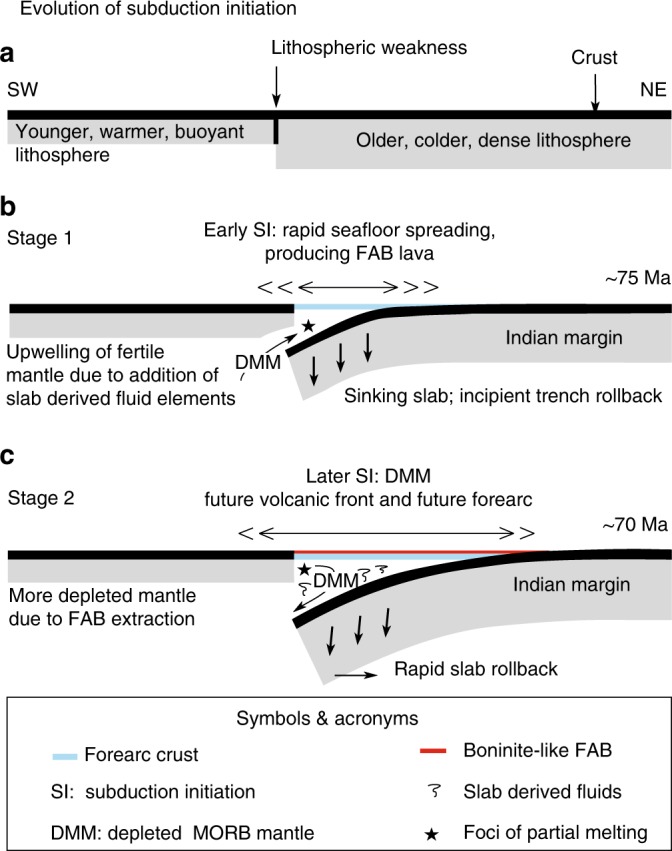
Conceptual model of intra-oceanic subduction initiation. The cartoon shows general evolution of SI (modified after Metcalf and Shervais^71^). **a** SI nucleates at a lithospheric weakness, e.g. a transform fault, which separates older, colder, denser lithosphere from younger, warmer, buoyant lithosphere^31,41,47,48,50^. **b** Incipient sinking of the older lithosphere instigates seafloor spreading in the proto-forearc. Partial melting of fertile, MORB-like mantle produces forearc basalts (FAB). **c** Based on observed distinct geochemical imprints in LB, SI appears to have been aborted during Stage 2. A slab rollback faster than the plate convergence results in extension on the upper plate and ultimate termination of slab pull^66^. Extraction of FAB leaves a depleted MORB mantle (DMM), which is subsequently tapped during Stage 2 with culmination of boninites (BON) or Boninite-like rocks. Subduction, if continued past Stage 2 would have lead to production of a magmatic arc as observed in the Izu–Bonin Marianas and other intra-oceanic arc systems^41,47,48^

Discovery of relict SI in the LB questions conventional rift models, especially with no such prior knowledge and therefore, demands an in-depth corroboration. Insights from the IBM forearc, one of the most extensively studied oceanic forearcs, have led to a paradigm shift in understanding how SI occurs^31,41,42^. Robust numerical simulations^50–52^ suggest that besides collisional tectonics (resulting in induced SI^47^), extensional failures along potential weak zones (transform faults/fracture zones/detachment surfaces) or passive margin collapse could spontaneously trigger incipient SI^47,48^. Pioneering works of Stern^47^ distinguished between induced and spontaneous SI on the basis of whether plate convergence leads to lithospheric failure or vice-versa. To date, there are no known occurrences of a Cenozoic SI caused by passive margin collapse^47^. It is widely argued that the lithospheric strengths of transitional crusts adjoining passive margins can be quite variable depending upon the degree of magmatism and presence (or absence) of crustal faults^41,47^. Thus, potential weak zones (e.g., transform faults/fracture zones) could become highly plausible targets for SI^47,50,51^ along passive margins. In view of this, we explore one of the most robust tectonic models for SSZ formation^47,48^ to assess the genesis of the LB (Fig. 6). According to this model, SI is likely when two lithospheric blocks of different ages and density are abutted against each other, creating a potential weak zone. In this situation, the older, denser oceanic lithosphere spontaneously sinks into its underlying asthenosphere (Fig. 6). Asthenosphere beneath the upper plate adjacent to the sinking rises rapidly into the gap left as the denser lithosphere sinks. Initially, MORB-like magmas are produced through the partial melting of a fertile, lherozolite source in the upper plate. Lava compositions are initially MORB-like in most respects, but may show an arc-like depletion in HFSE and enrichments in LILE thereafter. Eventually, a faster slab rollback than the plate convergence could lead to potential extension in the upper plate (Fig. 6) and thereby abortion of subduction .

Knowledge of intra-oceanic subduction during the late Cretaceous along the leading edge of the Indian margin is not new as evident from Neotethyan ophiolitic emplacement along its northern and western edges^15–17^ (Fig. 5). Contemporaneous ophiolitic exposures on eastern Arabian margin^15,17,43–46^ affirm that the boundary between the two plates was not only transcurrent but also intermittently convergent^15,17^. This essentially suggests that divergent and convergent phases must have frequently alternated in-tune with the changing plate motions^15–17^. The concomitant tectonic scenario around the western margin of India during the late Cretaceous was therefore conducive for generation of an incipient arc and SSZ type crust (Fig. 5). Furthermore, the early-late Cretaceous paleogeographic configuration^20,21,23,24^ of the Indian Ocean (Fig. 5) strongly favours lithospheric juxtapositions of contrasting ages and density across possible weak zones in the proto-LB^7,20–24^ (Fig. 2). Concurrent plate rotations^23,24^ accompanied oblique and asymmetric extension^18–21^ (Fig. 5), as evident from magnetic lineations in the proto-Gop rift-LB (~79–67 Ma)^18–20^. Ample geochemical and geo-tectonic constraints enable us to argue that prolonged rotational compression along the leading edge of the Indian margin must have activated SI and proto-forearc magmatism in the LB in sync with SSZ/island arc formation and ophiolite emplacement in the north (Fig. 5). Previous studies^15–17,23^ also envisioned possible seaward continuation of compressional tectonics into the Arabian Sea adjoining proto-Indian margin. Plate kinematic modelling supports that the NW Indian margin was contiguous to the Seychelles–India composite block^15–17,23^ during the early Cenozoic. We propose that concurrent plate instabilities may have provided the requisite impetus for spontaneous SI while the LB crust was still young. In view of the prevailing tectonic scenario (Fig. 5) we further opine that rapid slab rollback and associated collisional events^17,43,44^ that terminated subduction in the northern edge (e.g. Muslim Bagh-Bela SSZ environment) was also responsible for termination of SI in the LB.

Thermo-mechanical modelling supports that incipient SI may occur rather spontaneously (as quickly as ~0.3–5 Myr) to become either a self-sustaining or a failed process depending upon ambient plate stresses^47,48^. It is equally important to recognise that extensional tectonics within an overall rotational framework could play a decisive role in restraining slab pull^47^ (Fig. 6). We favour a similar mechanism to support nascent SI in the LB. Bhattacharya et al.^18–20^ proposed that seafloor spreading in the LB occurred 79–67 Ma^18–20^. Based on magnetic anomalies^18^ and biostratigraphic age constraints from early Paleocene sediments immediately overlying igneous basement at Site U1457^26^ (Fig. 2), we hypothesise that nascent SI and subsequent rifting in the LB must have occurred prior to ~63 Ma. Eventually, after arrest of SI, rifting might have moved out of the LB to the west of the LR that separated Seychelles from the LR and heralded the modern Carlsberg ridge (Fig. 5). Importantly, under such a scenario, locating relicts of a trench preserved in LB is highly unlikely today. Whether a remnant slab underlies the LB or delaminated (slab break-off), would need to await detailed tomographic analysis.

Collective consideration of the petrological, geochemical and isotopic characteristics summarised above suggests that the Laxmi basin has a distinct forearc crustal affinity. This includes common occurrence of highly calcic plagioclase and typical olivine–clinopyroxene–plagioclase crystallisation sequence instead of a typical MORB (plagioclase before clinopyroxene) sequence; enrichments in LILEs, LREEs and depletion in HFSEs relative to N-MORB in response to aqueous fluids and melts expelled from the subducting slab; higher oxygen fugacity than N-MORB, evident from lower arc-like Ti/V ratios; and enriched radiogenic ^87^Sr/^86^Sr, and depleted ^143^Nd/^144^Nd ratios, in the volcanic rocks relative to MORB. The above direct geochemical indicators point towards a transient SI phase in the LB. New drilling and coring data from Site U1457 for the first time demonstrate that the evolution of the northern Indian Ocean was far more complex than previously thought. Using decisive geochemical and isotopic signatures and other supporting geophysical observations we envisage that the LB crust has an unambiguous forearc affinity. We acknowledge the fact that future in-depth multi-disciplinary studies could yield additional corroborative evidence about the geochemically inferred SI event in the LB, especially in a regional context. This would include detailed structural and stratigraphic records as well as high precision radiometric dating of the basement rocks. Nevertheless, the fundamental characterisation of the LB crust, which was the primary objective of this work, would remain unaffected by prospective ancillary investigations. Importantly, results presented here possibly mark the first ever evidence of SI along a passive margin during the Cenozoic. The findings from the LB defy prevailing hypotheses about the genesis and early evolution of the Indian Ocean. While we continue to evaluate further ramifications of the unexpected outcomes, the crucial discovery reported here could have far-reaching implications in terms of final Gondwana dispersal, pre-collisional convergence history of India and Eurasia as well as subduction initiation along abutting passive margins.

## Methods

### Multichannel seismic data acquisition and processing

Around 5000 km long 2D seismic profiles were acquired by the ESSO-National Centre for Polar and Ocean Research (NCPOR) using a 6 km long streamer, with a recording length of 12 s two-way travel time (TWT) and 50 m shot spacing. Location of these seismic profiles are shown in Fig. 1. The location of the drill Site U1457 on the seismic line is marked in Fig. 2. Various morphological as well as sub-seafloor features in the basin are successfully imaged using seismic as well as high resolution bathymetry data around IODP drill sites in LB. Seismic data were processed using a standard approach involving multiple removals and pre-stack time migration. Processed time seismic sections were depth-converted using interval velocities derived from stacking velocities. Considering high data quality and constrained velocity analyses, uncertainties in time-depth conversion are estimated to be within ±10%. Additional stratigraphic constraints are obtained from published literature and deep boreholes^26,58,59^.

### Whole rock geochemistry

X-ray fluorescence (XRF) spectrometer (Philips PW-2440 Magix-PRO) attached with fully automated 100 KV X-ray generator, microprocessor controller and automatic sample changer at National Geophysical Research Institute, Hyderabad, India was used to determine the major oxides of representative basalt samples. Two grams of the powdered sample was weighed using an analytical balance with a precision as low as 0.0001 g. Pressed pellets were prepared using collapsible aluminium cups^56^. These cups were filled at the bottom with ~2.5 g of boric acid, and 2 g of the fine powdered sample was placed on top, and then pressed under a hydraulic pressure of 25 tons to obtain the pellet of each sample. International rock reference materials from the US Geological Survey (BHVO-1, BCR-1, BIR-1), Geological Survey of Japan (JB-1, JB-1a, JB-2, JB-3) and Chinese reference material (GSR-3) were used to prepare the calibration curves for major and trace elements, and to check the accuracy of analytical data. The precision obtained for most of the major oxides were <2% RSD.

### Trace element determination

Trace elements including rare earth (REE) and high-field strength elements (HFSE) were determined in solutions prepared from homogenised sample powder dissolved in reagent grade HF:HNO_3_ acid mixture in Savillex® screwtop vessels. A test portion (0.05 g) of sample was added to 25 ml Savillex® Teflon pressure decomposition vessels. To each sample, 10 ml of an acid mixture (containing 7:3 HF-HNO_3_) was added. Subsequently, 5 ml of 1 ng/ml ^103^Rh solution was added as an internal standard to each Savillex® vessel. After thorough swirling, the vessels were tightly closed and kept on a hot plate at ~140 °C for 48 h. Following this, the vessels were opened and the contents were evaporated at 200 °C to near dryness with a few drops of HClO_4_ to ensure complete removal of HF from the mixture. It was further dissolved by adding 10 ml of 1:1 HNO_3_ and the volume was made to 250 ml with Milli-Q® de-ionised water (18 MΩ), and the solution was stored finally in HDPE bottles. Matrix matching certified reference materials BHVO-1, BCR-1 (USGS), JB-2 (Japan) along with a couple of procedural blanks were also prepared with the sample batch by adopting the same protocol described above to negate errors due to reagent and handling. In the present investigation, very clear solutions were obtained for all the samples and calibration standards. Solutions were analysed at CSIR-NGRI, Hyderabad, by high resolution inductively coupled mass spectrometer (HR-ICP-MS) (Nu Instruments Attom®, UK) in jump-wiggle mode at moderate resolution which permits all the analytes of interest to be measured accurately^57^. The sample introduction consisted of a standard Meinhard® nebuliser with a cyclonic spray chamber housed in Peltier cooling system. All quantitative measurements were performed using the instrument software (Attolab v.2.6.0), while the data processing was done using Nu Quant®, which uses knowledge-driven routines in combination with numerical calculations (quantitative analysis) to perform an automated/manual interpretation of the spectrum of interest. Instrument was optimised using a 1 ppb tuning solution and the sensitivity of ^114^In was about 1 million cps. Oxide and oxy-hydroxide ratios were low (<0.2%) and the double charges ions ratio was <3%. Mass bias fractionation and several well-known isobaric interferences were addressed by using certified geochemical reference materials. Precision and accuracy are better than 3% RSD for the majority of trace elements.

### Radiogenic isotope analyses

The Sr and Nd isotopic analyses of 17 rock samples were carried out at the National Geophysical Research Institute (NGRI), Hyderabad, India, following well-established protocols^60,61^. Whole rock powders were leached with 10 mL of 6 M ultrapure HCl in acid cleaned centrifuge tubes. The leaching step was repeated until the supernatant was transparent, and colourless. The residue was subsequently rinsed with Milli-Q water to eliminate any trace of acid, centrifuged and dried down overnight at 100 °C. The Sr and Nd isotope ratios were measured using a Nu Plasma^TM^ MC-ICPMS in static multi-collection mode. Sample solutions of Sr and Nd were prepared in 2% (v/v) optima HNO_3_. Sr and Nd isotope ratios were corrected for mass fractionation using ^86^Sr/^88^Sr = 0.1194 and ^146^Nd/^144^Nd = 0.7219. The average values for NIST SRM987 and JNdi-1 during the course of two analytical sessions are ^87^Sr/^86^Sr = 0.710293 ± 38 (*n* = 7) and ^143^Nd/^144^Nd = 0.512065 ± 18 (*n* = 7) at the 2σ level of uncertainty. The in-run precision (2SE) was within the 2σ external reproducibility. Few samples analysed for Nd isotopes have higher than the 2σ external error for which combined error was considered. Measured ratios were normalised to the accepted ratio of 0.710245 for NIST SRM987 ^87^Sr/^86^Sr and 0.512115 for the JNdi-1 ^143^Nd/^144^Nd^62^. Epsilon Nd values were calculated using chondritic value of ^143^Nd/^144^Nd = 0.512638^63^. USGS Basalt standard BCR-2 was used to monitor the analytical quality, yielding ratios ^87^Sr/^86^Sr = 0.705048 ± 29 and ^143^Nd/^144^Nd = 0.512643 ± 16, which are within the recommended values of analytical errors^64,65^. The total procedural blanks were <150 pg for Sr, and ~35 pg for Nd insignificant compared with the size of the samples analysed.

## Supplementary information

Supplementary Information

## Data Availability

The data that support the findings of this study are available from the corresponding author (D.P.) upon reasonable request.
