# Supplementary material for: Relict subduction initiation along a passive margin in the northwest Indian Ocean
Source: Nat Commun. 2019 May 21;10:2248. doi: 10.1038/s41467-019-10227-8 (PMC6529441; doi:10.1038/s41467-019-10227-8)
Supplement: Supplementary file 1 — Supplementary Information [file 41467_2019_10227_MOESM1_ESM.pdf]

Supplementary Information

for

Relict subduction initiation along a passive margin in the northwest Indian Ocean

Pandey et al.

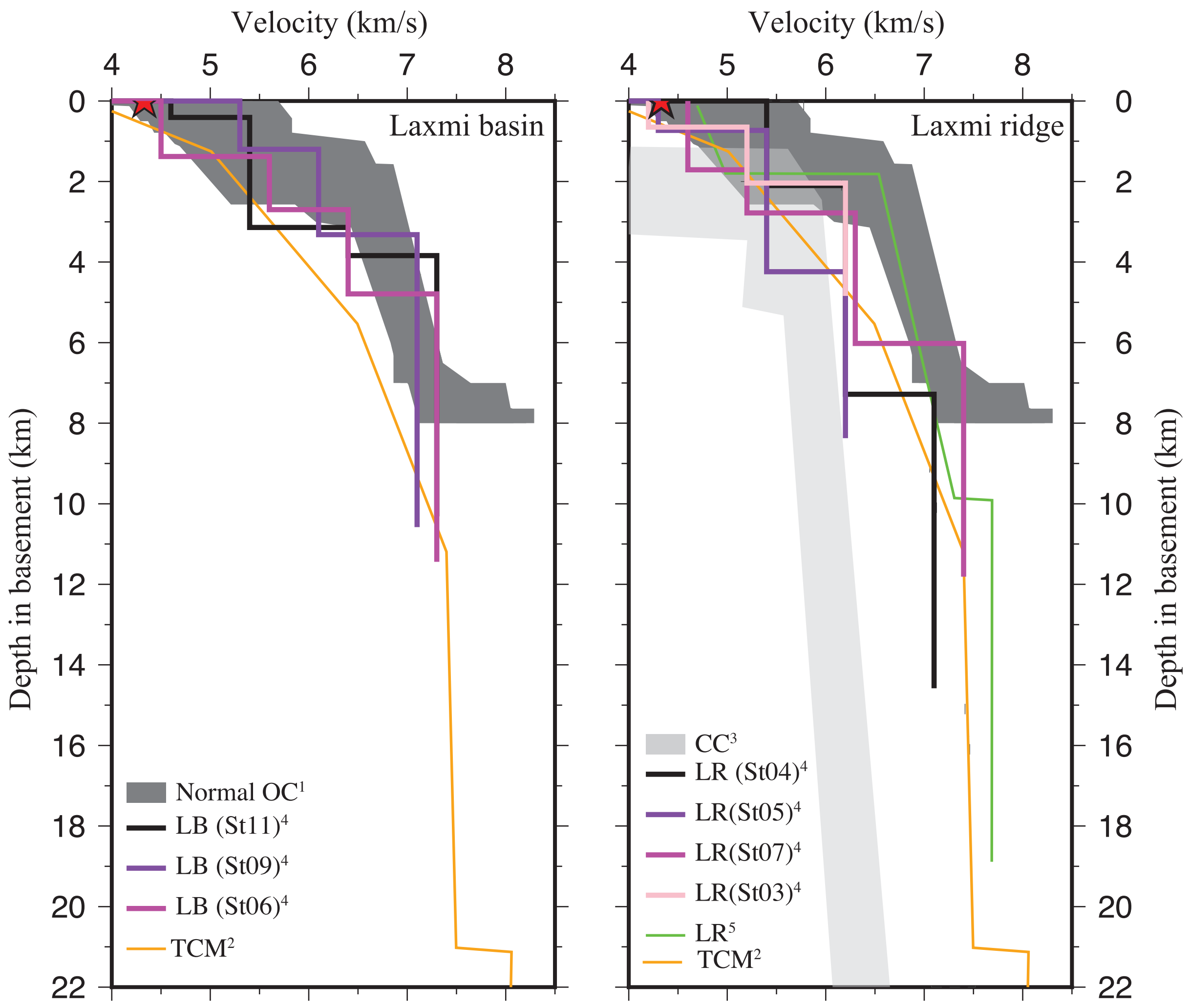

(a)

(b)

Suplementary Figure 1: Comparison of seismic velocity-depth profiles beneath Laxmi basin (a) and Laxmi ridge (b) with various known tectonic settings. The dark grey bounds represent average oceanic crust (59-127 Ma) velocities[1]. The light grey bounds mark velocities for extended continental crust[2] and orange line represent thinned continental margin (TCM)[3]. Published 1-D velocity-depth models from different refraction stations[4,5] are shown on the plots.

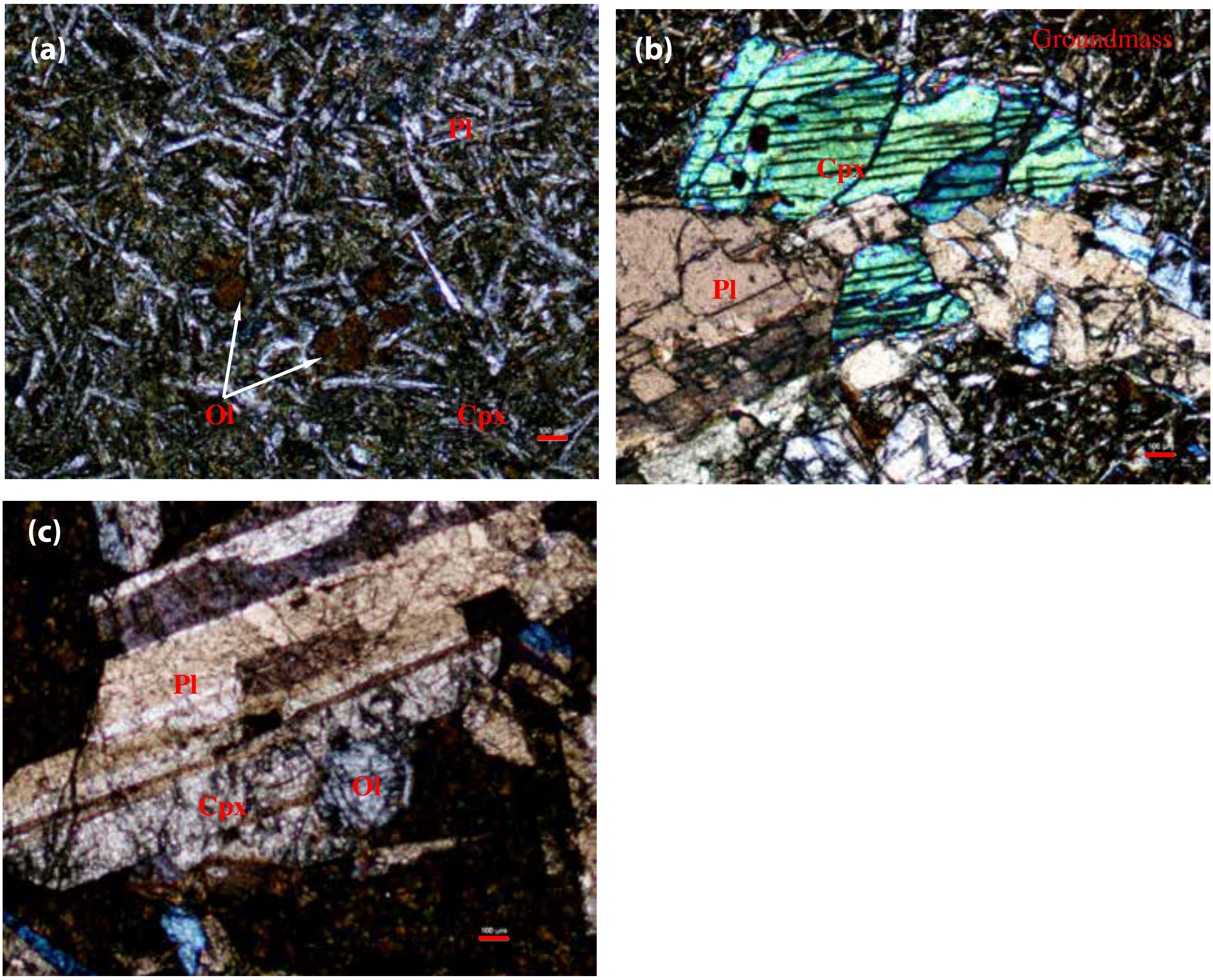

Suplementary Figure 2: Thin section microphotographs of Site U1457 basalts from Laxmi basin in cross-polarized light showing various characteristic texture. (a):355U1457-97R1-16/20; Aphyric lava with microlites of plagioclase, clinopyroxene and altered olivine showing intersertal texture. (b): 355U1457-97R2-60/70; Phyric lava with glomerocrysts of clinopyroxene, plagioclase and olivine set in a groundmass of microlitic plagioclase and clinopyroxene (c): 355U1457-96R3-15/19; mega-plagioclase laths encapsulating olivine and clinopyroxene. The scale bar=100 microns.

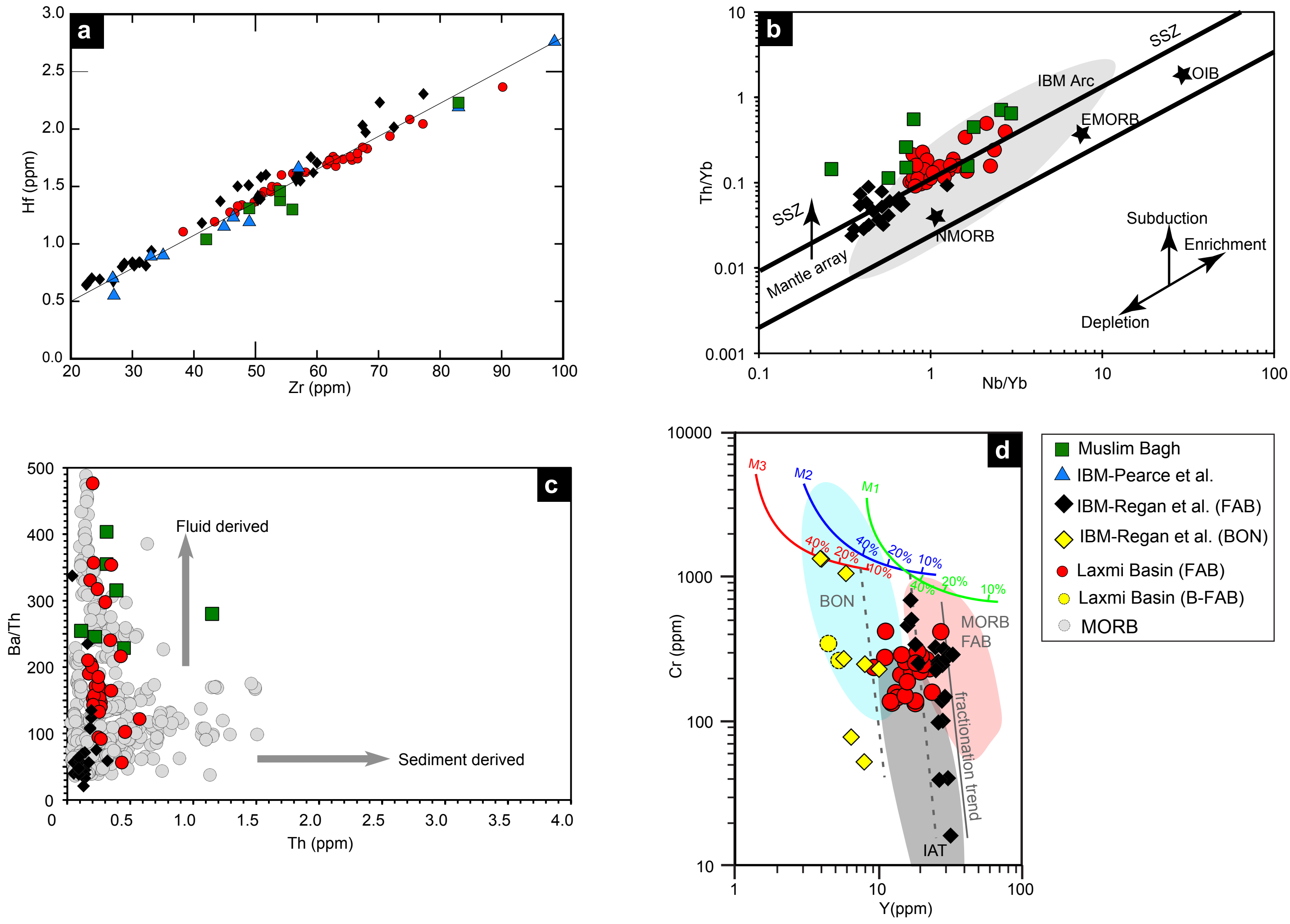

Suplementary Figure 3: Geochemical classifications of LB crust (a) The covariance diagram between refractory element pair Zr-Hf for the LB lava demonstrating strong positive correlation[6,7] . Their constant ratios, in consonance with other arc settings, rule out their role in fractionation during petrogenesis. Data from other tectonic settings are also plotted for comparison; (b) The Nb/Yb-Th/Yb discriminant diagram[8,9]. The MORB and OIB domains form a diagonal mantle array on this diagram, whereas magmas having subduction components are displaced towards higher Th/Yb values. While few of LB basalts plot within the mantle array, majority of the samples are displaced toward higher Th/Yb ratios characterizing the LB lava with clear subduction influence; (c): Plot between Ba/Th ratio and Th abundances for LB lava to evaluate role of hydrous fluids versus sediments during subduction process. High Ba/Th values with low Th abundances are widely attributed to preferential mobilization of LILEs (e.g Ba) through hydrous fluids components (vertical array) as opposed to the sediment flux (horizontal array) from the downgoing slab; (d) The Cr-Y discrimination diagram to assess the extent of fractionation and degree of partial melting for LB basalts[9]. Mantle source compositions and incremental batch melting paths for reference. Petrogenetic modelling using a compatible (Cr) versus an incompatible (Y) element demonstrates that most of the LB basalts can be attributed to partial melting of a much depleted source. Various symbols as labelled. Abbreviations: SSZ – Supra-subduction zone; MORB- Mid Ocean Ridge Basalt; IBM-Izu-Bonin Marinana arc; Bon-Boninites; FAB – Forearc basalts.

Supplementary References

1. White, R.S., McKenzie, D. & O'Nions, R.K. Oceanic crustal thickness from seismic measurements and rare earth element inversions, *J. Geophys. Res.*, **97**(B13), 19683–19715 (1992).
2. Eldholm, O. & K. Grue, North Atlantic volcanic margins: Dimensions and production rates, *J. Geophys. Res*., 99, 2955-2968 (1994).
3. Christensen, N.I. & Mooney, W.D. Seismic velocity structure and composition of the continental crust: a global view. *J. Geophys. Res.* 100, 9761-9788 (1995).
4. Naini, B. R. & Talwani, M. Structural framework and the evolutionary history of the continental margin of western India. *AAPG Memoir* 34, 167–191 (1982).
5. Minshull, T.A., Lane, C.I., Collier, J.S., Whitmarsh, R.B. The relationship between rifting and magmatism in the Northeastern Arabian Sea. *Nat. Geosci.* **1**, 463–467 (2008).
6. Sun S. & McDonough W.F. Chemical and isotopic systematics of oceanic basalts: implications for mantle composition and processes. *Geol. Soc. Lond. Spec. Publ.* **42**, 313-345, (1989).
7. Noiret, G., et al. Is the Vourinos Complex an island arc ophiolite?, *Earth Planet. Sci. Lett.*, 56, 375-386 (1981).
8. Dilek, Y., & Furnes, H. Ophiolite genesis and global tectonics: Geochemical and tectonic fingerprinting of ancient oceanic lithosphere: *Geological Society of America Bulletin*, v. 123, p. 387–411. doi:10.1130/B30446.1 (2011).
9. Pearce, J.A., et al. Characteristics and tectonic significance of supra-subduction zone ophiolites. *Geol. Soc. Lond. Spec. Publ.* 16, 77–94. (1984).
